# Comparative Evaluation of the Translucency of Polyetheretherketone (PEEK) Veneered With Two Different Materials: An In Vitro Study

**DOI:** 10.7759/cureus.94224

**Published:** 2025-10-09

**Authors:** M P Chinmayi, Gautam Shetty, S M Kedar, Lokesh B Kanchan, Rohit S Kundu, Krishna Kumar U, Maria Jenifer

**Affiliations:** 1 Department of Prosthodontics, Rajarajeswari Dental College and Hospital, Bengaluru, IND; 2 Department of Prosthodontics, Crown and Bridge, ESIC Dental College, Kalaburagi, IND

**Keywords:** aesthetics, composite resin, contrast ratio, lithium disilicate, peek, spectrophotometric analysis, translucency parameter, veneer

## Abstract

Introduction: Modern dentistry seeks materials with enhanced aesthetics and properties for restorations. While polyetheretherketone (PEEK) is promising, its low translucency necessitates veneering for visible areas. Lithium disilicate glass-ceramic and composite resins are common veneering options. This in vitro study aimed to comparatively evaluate the translucency of PEEK when veneered with lithium disilicate glass-ceramic and composite resin. This is crucial for achieving predictable aesthetic outcomes.

Methods: 20 sandblasted PEEK specimens (10mm x 10mm, 0.6mm thick) were divided into two groups (n=10). Group A (lithium disilicate) comprised 0.5mm thick A2 shade lithium disilicate veneers, fabricated via lost wax pressing, which were etched, silanized, and luted to PEEK cores with translucent light-cure resin cement. In Group B (composite resin), PEEK discs received an opaquer, then 0.5mm thick A2 shade composite resin was injected into a mold to form the bi-layered specimen. The translucency parameter (TP) and contrast ratio (CR) of the veneered specimens were assessed using a UV-Vis-NIR spectrophotometer. Data normality was confirmed via the Shapiro-Wilk test. Independent samples t-tests were then performed to evaluate inter-group differences, with statistical significance set at p < 0.05.

Results: Statistically significant differences were found between the materials regarding translucency and masking ability (p<0.001 for both TP and CR). Lithium disilicate veneered PEEK (Group A) showed a significantly higher mean TP of 11.42±0.55 (95% CI: 11.03-11.82), indicating superior light transmission. In comparison, composite resin (Group B) had a mean TP of 10.42±0.29 (95% CI: 10.21-10.63). Furthermore, lithium disilicate displayed a statistically higher mean CR of 1.360±0.01 (95% CI: 1.35-1.37), suggesting greater masking ability, compared to the composite resin CR of 1.320±0.01 (95% CI: 1.31-1.33).

Conclusion: Lithium disilicate glass-ceramic, as a veneering material for PEEK, offers a statistically significant advantage in both simulating natural enamel’s translucency and providing better masking. This makes it a more suitable choice for highly aesthetic PEEK-based restorations in demanding zones.

## Introduction

Modern dentistry heavily relies on advanced materials offering biocompatibility, low plaque affinity, and aesthetic properties akin to natural teeth. These advancements are crucial for restoring dental defects and meeting the growing patient demand for natural-looking restorations [1]. Polyetheretherketone (PEEK), a high-performance polymer, has emerged as a promising alternative for fixed dental prostheses. It boasts excellent biocompatibility, high temperature resistance, superior fatigue properties, and a modulus of elasticity similar to human bone, providing a beneficial damping effect. Furthermore, PEEK is radiolucent, avoiding image artifacts in medical scans, and doesn't cause metal allergies [2].

However, PEEK's inherent opacity and greyish white color significantly limit its use as a monolithic restoration, making additional veneering essential for aesthetic success [3]. Dental veneers, fabricated using materials like resin composite or ceramics, effectively address patients' aesthetic requirements. Lithium disilicate, a glass-ceramic, is particularly popular for laminate veneers due to its exceptional aesthetic properties, strength, and bonding capabilities. Its high translucency is achieved by ensuring similar refractive indices between its crystalline and glassy phases. Composite veneers are also widely utilized, with their translucency influenced by factors like filler content and particle size.

Evaluating translucency is paramount for the aesthetic success of restorations. Previous studies have used spectrophotometers to assess translucency and opacity, often employing the contrast ratio (CR) and translucency parameter (TP) [4]. Despite PEEK's promising mechanical properties, there's limited data on the translucency of PEEK veneered restorations. This in vitro study aims to address this gap by evaluating and comparing the translucency of PEEK veneered with two different veneering materials.

## Materials and methods

This in vitro study was conducted in the Department of Prosthodontics Crown and Bridge, Rajarajeswari Dental College and Hospital, Bengaluru. A total of 20 PEEK specimens were prepared and divided into two groups (n=10) for evaluation.

Sample size calculation

The sample size for the present study was estimated using G*Power software (ver. 3.1.9.7; Heinrich-Heine-Universität Düsseldorf, Düsseldorf, Germany). The calculation was performed using the following parameters: a two-tailed α error of 5% (α=0.05), a study power of 80%, and an estimated effect size (d) of 1.35 (based on the mean ΔE difference between study groups reported by Fahmy et al. [1]. This analysis demonstrated that a minimum of 20 samples would be needed, resulting in 10 samples for each study group (n=10).

Specimen preparation

Standardization for PEEK Samples

**Figure 1:**
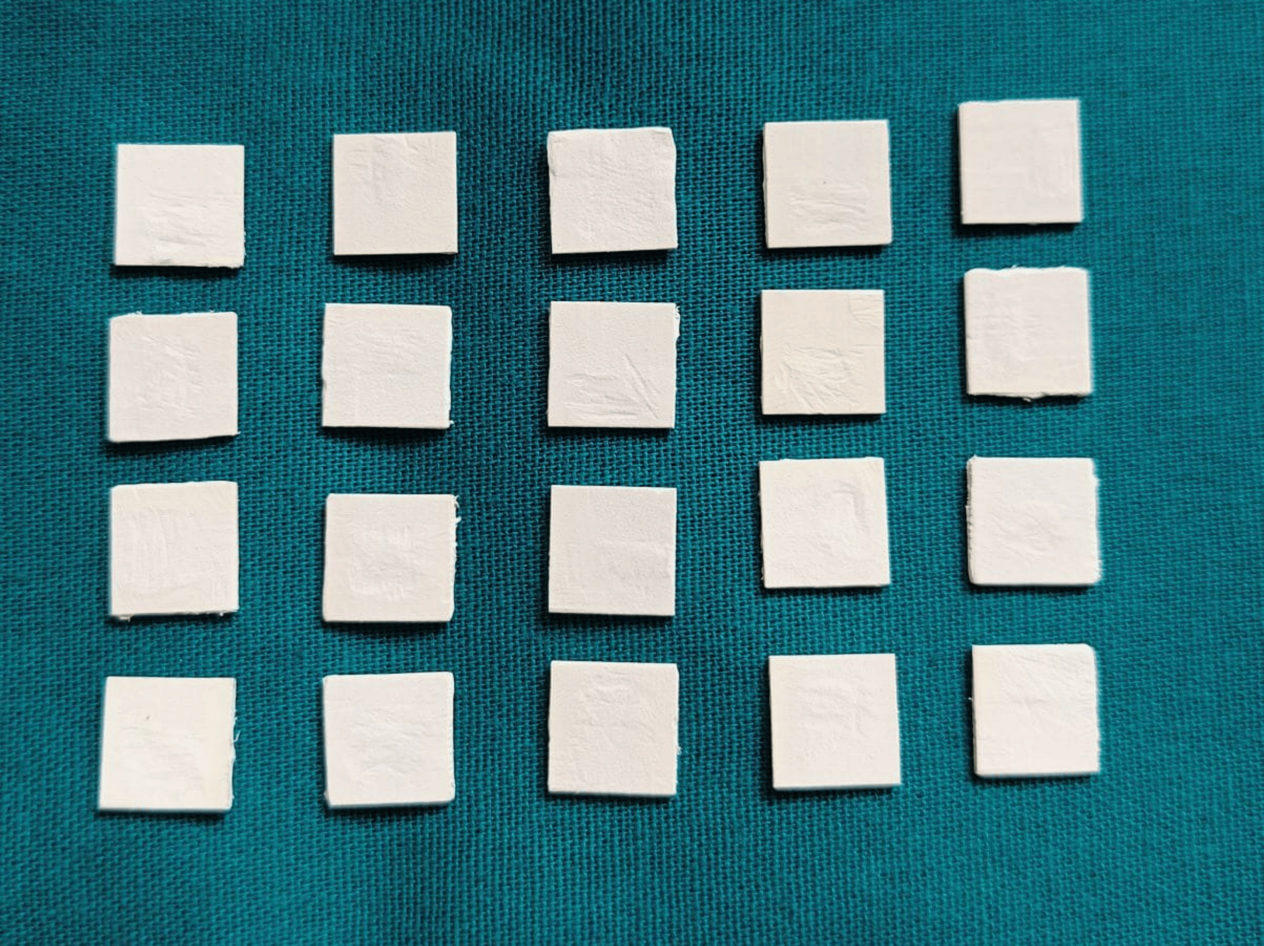
PEEK samples PEEK: polyetheretherketone

PEEK samples for this study were fabricated from breCAM.BioHPP PEEK discs (Bredent GmbH & Co KG, Germany). Digital designs (Exocad software) created square discs measuring 10mm x 10mm x 0.6mm (Figure 1). These STL files were milled from 98mm diameter, 16mm thick PEEK discs (shade DS2) using a 5-axis milling machine unit (Ivoclar Vivadent Pvt. Ltd., Schaan, Liechtenstein) with a dry milling technique. Post-milling, samples were detached and sandblasted with 110 µm aluminum oxide particles at 2.0 bar pressure for 10 seconds to ensure uniform surface roughness. Dimensions were verified using a digital caliper.

Standardization for Lithium Disilicate Samples (Group A)

**Figure 2:**
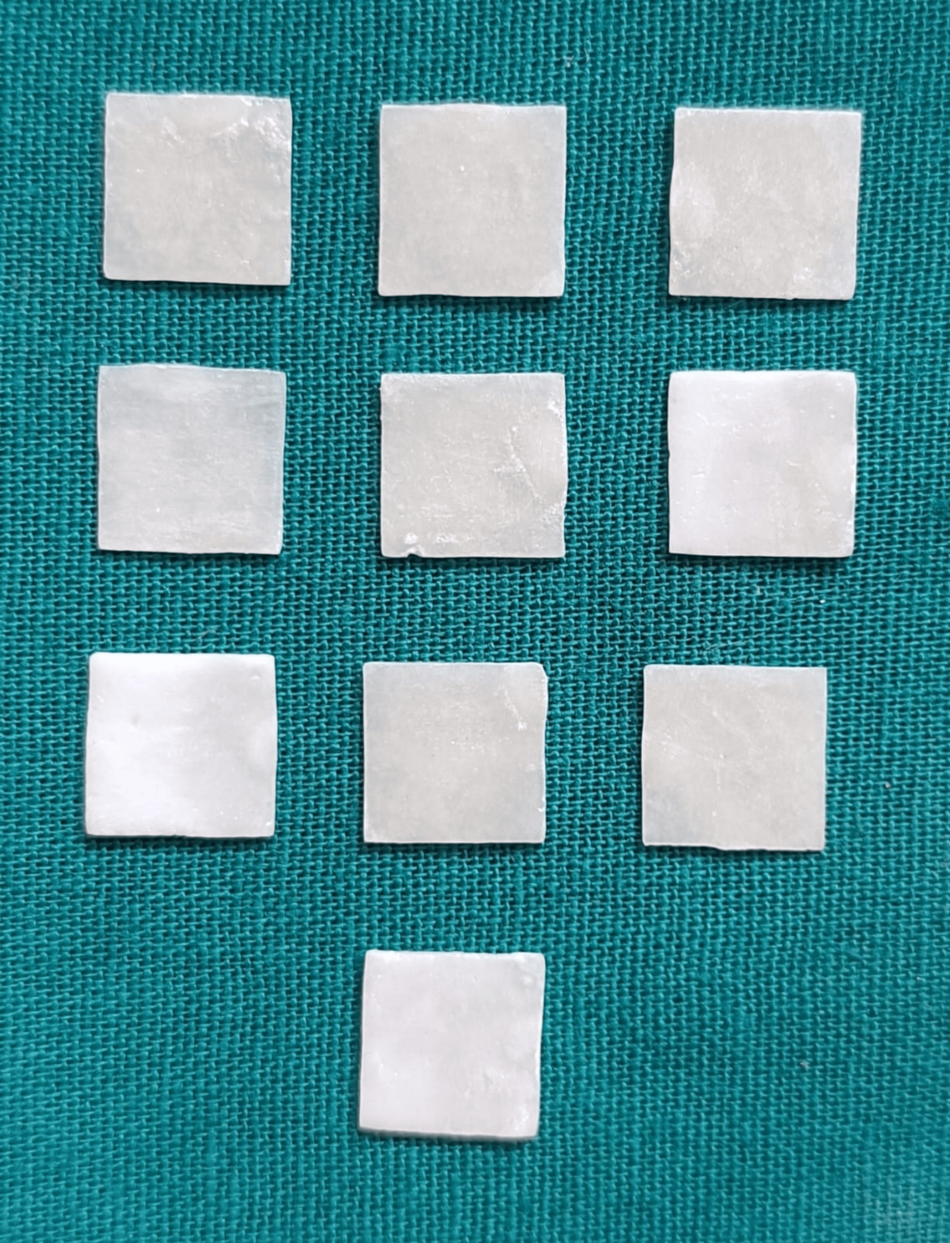
Lithium disilicate samples

Lithium disilicate veneer specimens (10mm x 10mm x 0.5mm) were fabricated from A2 shade, low translucency Upcera Press ingots (Shenzhen Upcera Dental Technology Co. Ltd., Shenzhen, China) (Figure 2). Digital designs (Exocad software, Darmstadt, Germany) were milled as wax patterns using a five-axis milling machine unit. These wax patterns were invested with IPS PressVEST Speed (Ivoclar Vivadent AG, Schaan, Liechtenstein). After burnout at 1562 °F, a cold lithium disilicate ingot was pressed into the hot investment ring using a Programat EP3010 press furnace (Ivoclar Vivadent Pvt. Ltd.). Samples were then cooled, divested with glass beads at 4 bar pressure, and ultrasonically cleaned. The reaction layer was removed with 110 microns A1203 at 2 bar pressure, and sprues were trimmed with a diamond disc. A total of 10 finished and glazed samples were confirmed for consistent thickness with a digital caliper.

Standardization procedure for cementation of lithium disilicate samples on PEEK samples: Each ceramic disc was etched with 9.5% hydrofluoric acid (Porcelain Etch, Ultradent Products, Inc., South Jordan, UT) for 10 seconds, rinsed, and a silane coupling agent (Ultradent Products) was applied for 60 seconds until a matte finish according to the manufacturer’s recommendation. PEEK and lithium disilicate discs were assembled in a special metal mold and luted with translucent light-cure resin cement (Relyx, 3M ESPE, St. Paul, MN). A 1 kg weight standardized pressure and full polymerization was achieved by light-curing for 90 seconds using a blue LED light with an intensity of ∼1000-1200 mW/cm2.

Standardization for Preparing Composite Resin Samples (Group B)

For this group, each PEEK disc in the metal mold received Empress Direct Opaque (Ivoclar Vivadent). Beautifil Injectable composite resin (Shofu Inc., Kyoto, Japan) of A2 shade was then injected to fill the mold, ensuring the bi-layered specimen met the mold's thickness. A 1 kg weight standardized pressure, followed by 90 seconds of light curing. A customized mold ensured uniform thickness and polymerization.

Preparation of metal mold: A special 10mm x 10mm x 1.5mm cobalt chromium metal mold was designed and prepared using a Metal 3D Printer (2oneLab GmbH, Germany) to standardize veneer sample formation and cement thickness.

Method of collection of data

Spectrophotometer Measurements

Reflectance (Y) and color coordinates (CIE Lab*) were measured using a Lamda 1050+ UV-Vis-NIR Spectrophotometer (PerkinElmer, Waltham, MA) under CIE illuminant D65, with a 2-degree observer function and zero-degree viewing geometry.

Translucency parameter (TP) was calculated as the color difference (ΔE) between the material over black and white backgrounds:

TP= $\sqrt{\left( L^{*}b-L^{*} w\right)^{2}+\left( a^{*} b-a^{*}w\right)^{2}+\left( b^{*} b-b^{*}w\right)^{2}}$

Contrast ratio (CR) was calculated as the ratio of reflectance over a black background (Yb) to that over a white background (Yw): CR=Yb/Yw. A CR of 0 signifies transparent, while 1 signifies opaque. The obtained values were tabulated for statistical analysis.

Statistical analysis

Statistical analysis was conducted using Jamovi (v2.6.44) and Statisty Online. Descriptive statistics, including mean, standard deviation, minimum, maximum, and 95% confidence intervals, were computed for Translucency Parameter (TP) and Contrast Ratio (CR) for both groups. Data normality was confirmed via the Shapiro-Wilk test (p > 0.05). Independent- samples t-tests were then performed to evaluate inter-group differences, with statistical significance set at p < 0.05.

## Results

This comparative study evaluated the translucency of PEEK veneered with lithium disilicate (Group A) and composite resin (Group B).

The Commission Internationale de l'Eclairage (CIE) recommended calculating color difference (ΔE) based on CIELAB color parameters, which was largely used to compare translucency among materials [1].

Translucency parameter (TP)

Individual TP values for PEEK veneered with lithium disilicate (Group A) and composite resin (Group B) are presented in Table 1. TP values were consistently higher for lithium disilicate, ranging from 10.42 to 12.51, compared to composite resin, which ranged from 9.80 to 10.86. Sample 9 showed the maximum TP for lithium disilicate, and Sample 2 for composite resin. Conversely, Sample 10 for lithium disilicate and Sample 4 for composite resin exhibited the minimum TP values. The mean TP for lithium disilicate was 11.42, and for composite resin, it was 10.42.

**Table 1:** Individual Translucency Parameter Values for PEEK Veneered With Lithium Disilicate (Group A) and Composite Resin (Group B) PEEK: polyetheretherketone

Sample No.	Translucency parameter (lithium disilicate - Group A)	Translucency parameter (composite resin - Group B)
1	11.52	10.47
2	11.11	10.86
3	11.33	10.43
4	11.42	9.80
5	11.35	10.52
6	11.40	10.43
7	12.01	10.18
8	11.14	10.72
9	12.51	10.48
10	10.42	10.29
Mean	11.42	10.42

Descriptive statistics for the TP are further detailed in Table 2. The mean TP for lithium disilicate (Group A) was 11.42 ± 0.55 (95% CI: 11.03-11.82), while composite resin (Group B) had a mean TP of 10.42 ± 0.29 (95% CI: 10.21-10.63). Lithium disilicate consistently exhibited higher TP values than composite resin, indicating its superior light transmission. The standard deviation for lithium disilicate (0.55) was higher than that of composite resin (0.29), suggesting greater variability within the lithium disilicate group.

**Table 2:** Descriptive Statistics of Translucency Parameters for Lithium Disilicate and Composite Resin PEEK samples PEEK: polyetheretherketone

Group	Mean	Standard deviation	Minimum	Maximum	95% confidence interval
Translucency parameter (lithium disilicate)	11.42	0.55	10.42	12.51	11.03 – 11.82
Translucency parameter (composite resin)	10.42	0.29	9.8	10.86	10.21 – 10.63

A comparison of the mean translucency parameter (TP) between the two groups is presented in Table 3. A p-value of <0.001 indicates a statistically significant difference between the two groups. This significant difference confirms that lithium disilicate allows for substantially greater light transmission compared to composite resin, reinforcing its higher translucency.

**Table 3:** Comparison of Mean Translucency Parameters Between Lithium Disilicate and Composite Resin Groups Using Independent Samples t-test

Material	Mean ± Standard Deviation	t-value	p-value
Lithium disilicate	11.42 ± 0.55	5.09	< 0.001
Composite resin	10.42 ± 0.29		

Contrast ratio (CR)

Individual CR values for PEEK veneered with lithium disilicate and composite resin are shown in Table 4. The CR values for lithium disilicate were marginally higher in nearly all samples. Sample 7 showed the maximum CR for lithium disilicate (1.377), and Sample 2 for composite resin (1.337). Sample 10 exhibited the minimum CR for lithium disilicate (1.322), while Sample 4 showed the minimum for composite resin (1.301). The mean CR for lithium disilicate was 1.360, and for composite resin, it was 1.320, indicating better light transmission with slightly more effective masking for lithium disilicate; Table 5 provides the descriptive statistics for these CR values. The mean CR for lithium disilicate was 1.360 ± 0.01 (95% CI: 1.35-1.37), while for composite resin, it was 1.320 ± 0.01 (95% CI: 1.31-1.33). Lithium disilicate consistently exhibited higher CR values than composite resin, indicating its greater masking ability or opacity. Both groups showed relatively small standard deviations (0.01 for both), suggesting consistency within each material.

**Table 4:** Individual Contrast Ratio Values for PEEK Veneered With Lithium Disilicate and Composite Resin PEEK: polyetheretherketone

Sample No.	Contrast Ratio (Lithium Disilicate)	Contrast Ratio (Composite Resin)
1	1.365	1.320
2	1.349	1.337
3	1.359	1.323
4	1.362	1.301
5	1.359	1.326
6	1.361	1.324
7	1.377	1.314
8	1.347	1.334
9	1.355	1.324
10	1.322	1.318
Mean	1.360	1.320

**Table 5:** Descriptive Statistics of Contrast Ratio for Lithium Disilicate and Composite Resin PEEK Samples PEEK: polyetheretherketone

Group	Mean	Standard Deviation	Minimum	Maximum	95% Confidence Interval
Contrast ratio (lithium disilicate)	1.36	0.01	1.32	1.38	1.35 – 1.37
Contrast ratio (composite resin)	1.32	0.01	1.3	1.34	1.31 – 1.33

The comparison of the mean CR between the two materials is presented in Table 6. A p-value of <0.001 indicates a statistically significant difference between the two materials, indicating the superior masking ability of lithium disilicate compared to composite resin.

**Table 6:** Comparison of the Mean Contrast Ratio Between Lithium disilicate and Composite Resin Groups Using Independent Samples t-test

Material	Mean ± Standard Deviation	t-value	p-value
Lithium disilicate	1.36 ± 0.01	8.95	< 0.001
Composite resin	1.32 ± 0.01		

The Shapiro-Wilk test results (Table 7) showed that both groups followed a normal distribution for the two variables tested. For the translucency parameter, lithium disilicate (Group A) had W=0.9242, p=0.3937, and composite resin (Group B) had W=0.9362, p=0.5116. For the CR, the lithium disilicate (Group A) recorded W=0.8911, p=0.1744, while the composite resin (Group B) showed W=0.9457, p=0.6182. Since all p-values were greater than 0.05, the null hypothesis of normality was not rejected, indicating that the data in both groups can be considered normally distributed.

**Table 7:** Shapiro-Wilk Test for Normality of Translucency and Contrast Ratio for Lithium Disilicate and Composite Resin

Variable	Group	W statistic	p-value
Translucency	Lithium disilicate (A)	0.9242	0.3937
	Composite resin (B)	0.9362	0.5116
Contrast ratio	Lithium disilicate (A)	0.8911	0.1744
	Composite resin (B)	0.9457	0.6182

## Discussion

PEEK, a high-performance thermoplastic polymer, has transitioned from industrial to biomedical and dental fields since the 1980s. Initially valued for mechanical properties, chemical resistance, and high-temperature stability, PEEK's biomedical potential was recognized in the late 1990s. Its elastic modulus, closely approximating human bone and dentin, made it an attractive alternative for orthopedic and spinal implants by minimizing stress shielding. PEEK's dental integration offered biocompatibility, lightness, and a neutral, tooth-colored appearance, presenting a promising alternative to metal-based restorations for frameworks and implant components [5].

Despite PEEK's biomechanical advantages, its inherent low translucency and opaque appearance limit aesthetic dental restorations, especially in visible areas [3,6]. Consequently, PEEK frameworks require veneering with materials like lithium disilicate or composite resins for acceptable aesthetics [5,6]. Lithium disilicate, adaptable with diverse shades, is frequently chosen for veneering PEEK to mask aesthetic limitations [7,8]. This combination leverages PEEK's biomechanical benefits with essential aesthetic appeal [9]. Composite resin is also selected for PEEK substructures for its tooth-colored covering and moldability; however, it has comparatively lower long-term color stability and wear resistance than lithium disilicate [10].

This study's rationale stemmed from the critical need for optimal aesthetics in PEEK-based restorations. Given PEEK's opacity, veneering material selection is paramount for the final aesthetic outcome, particularly translucency and masking ability. Previous research indicated that even 1.5mm veneered PEEK might yield unacceptable color differences and lower translucency [3]. Translucency of dental restoratives is inversely influenced by thickness [11-13]. For lithium disilicate, clinicians can adjust veneer thickness to control light transmission and mask the opaque PEEK substructure [11,12,14]. Similarly, composite resin's translucency decreases with thickness, necessitating careful consideration to conceal dark backgrounds like PEEK [15-17]. Thus, this study compared the translucency and masking ability of PEEK veneered with lithium disilicate and composite resin at a standardized 0.5mm veneering thickness, providing insights into their optical performance.

Translucency is paramount in aesthetic dental restorations, crucial for mimicking natural tooth appearance. Natural tooth enamel and dentin exhibit varying translucency, allowing light to pass through while scattering it, essential for subtle depth and harmonious integration [13,18]. Without it, restorations appear opaque or lifeless.

TP and CR objectively quantify translucency. TP measures material translucency at a specified thickness, calculated as CIELAB color difference over black and white backgrounds [13,15]; higher TP means greater translucency. CR indicates opacity, defined as the ratio of luminous reflectance over black to white backgrounds [13]. Lower CR indicates higher translucency. Both are crucial for material selection [18,19].

This in vitro study evaluated the optical properties of PEEK veneered with lithium disilicate and composite resin. A total of 20 PEEK discs (10x10x0.6mm) were prepared for each group. For Group A, 10 lithium disilicate samples (0.5mm) were bonded to PEEK with 0.4mm resin cement, totaling 1.5mm thickness. For Group B, composite resin (A2 shade) was injected onto PEEK to a 0.5mm thickness.

Study results showed clear and statistically significant differences. Lithium disilicate had consistently and substantially higher TP values compared to composite resin (p<0.001). Furthermore, CR values were also significantly higher for lithium disilicate compared to composite resin (p<0.001), a difference reinforced by non-overlapping 95% confidence intervals.

The findings provide clear clinical guidance for selecting veneering materials for PEEK restorations. The significantly higher mean TP (11.42) and CR (1.36) of lithium disilicate compared to composite resin confirm its superior optical performance. This dual capability - high translucency for mimicking the natural vitality of enamel, and effective light scattering (indicated by higher CR) for masking the opaque PEEK core - makes lithium disilicate the preferred material for PEEK-based restorations in the aesthetic zone [10,13,14,20]. Clinicians should recognize that utilizing lithium disilicate, even at a standardized minimal thickness of 0.5 mm, offers a more predictable and aesthetically pleasing outcome.

Conversely, while composite resin (TP=10.42; CR=1.32) remains a viable, tooth-colored option for veneering PEEK, its statistically lower optical values suggest a compromise in the final aesthetic result, potentially leading to a more opaque or less lifelike restoration. This material may be more suitable for restorations in less visible areas or when budgetary or temporary needs outweigh the requirement for peak aesthetic integration. Therefore, the data support prioritizing lithium disilicate for PEEK restorations in aesthetically demanding cases, leveraging its inherent material science to overcome the core's opacity and achieve superior natural-looking results.

Study limitations and future research

This in vitro study's findings are subject to limitations, including its controlled laboratory setting, fixed material thicknesses, and restricted optical property assessment (lacking opalescence/fluorescence). Only a single type and shade of resin cement was used, which may influence optical outcomes due to variations in refractive indices among different cements. The study also did not incorporate long-term aging, staining, or evaluation across multiple shades or brands, limiting direct clinical translation. Future research should prioritize in vivo clinical trials, longitudinal aging and staining studies, comprehensive optical property evaluations, investigations into varied material thicknesses and designs, and explore the impact of different luting cements, mechanical properties, alternative surface treatments, and diverse PEEK compositions to provide a more holistic understanding.

## Conclusions

Within the limitations of this in vitro study, it was concluded that lithium disilicate glass- ceramic, when used as a veneering material for PEEK, exhibited a significantly higher mean translucency parameter. Conversely, composite resin, as a veneering material for PEEK, showed a significantly lower mean translucency parameter. The enhanced translucency and superior masking ability of lithium disilicate position it as a more suitable and advantageous material for achieving highly aesthetic outcomes in PEEK-based restorations.
